# Late Onset Rheumatoid Arthritis

**DOI:** 10.14336/AD.2025.0898

**Published:** 2026-10-24

**Authors:** Zhao Peng, Wenjing Liu, BinYu Huang, Qian Wang, Xinping Tian, Sean X. Leng, Lin Kang

**Affiliations:** ^1^Department of Internal Medicine, Peking Union Medical College Hospital, Chinese Academy of Medical Sciences & Peking Union Medical College, Beijing, China.; ^2^Department of Geriatrics, Peking Union Medical College Hospital, Chinese Academy of Medical Sciences & Peking Union Medical College, Beijing, China.; ^3^Department of Geriatrics, Gansu Province Second People's Hospital, Lanzhou, China.; ^4^Department of Rheumatology and Clinical Immunology, Chinese Academy of Medical Sciences & Peking Union Medical College, National Clinical Research Center for Dermatologic and Immunologic Diseases (NCRC-DID), Ministry of Science & Technology, State Key Laboratory of Complex Severe and Rare Diseases, Peking Union Medical College Hospital (PUMCH), Key Laboratory of Rheumatology and Clinical Immunology, Ministry of Education; No.1 Shuaifuyuan, Dongcheng District, Beijing, China.; ^5^W. Harry Feinstone Department of Molecular Microbiology and Immunology, Johns Hopkins University Bloomberg School of Public Health, Baltimore, MD, USA.; ^6^Division of Geriatric Medicine and Gerontology, Department of Medicine, Johns Hopkins University School of Medicine, Baltimore, MD, USA.

**Keywords:** Late onset rheumatoid arthritis, innate immune, adaptive immune response, age-related B cells

## Abstract

Late-onset rheumatoid arthritis (LORA) refers to rheumatoid arthritis (RA) with initial symptoms and signs appearing after the age of 60 or 65. A higher frequency of HLA-DRB1*01:01, *04:03, and *14:02 alleles and a lower frequency of HLA-DRB1*04 are observed in patients with LORA compared to young-onset RA (YORA). Due to immunosenescence, the immune response in LORA differs from that in YORA. Specifically, in LORA, there is an increase in *M1* macrophages and CD56^dim^ NK cells, whereas the numbers of *M2* macrophages, Mer proto-oncogene tyrosine kinase, and CD56^bright^ NK cells are reduced. Elevated levels of age-related B cells in older individuals may contribute to more swollen and tender joints, as well as higher disease severity scores in LORA than in YORA. Additionally, impaired DNA repair mechanisms, an increased ratio of CD4^+^/CD8^+^ T cells, and elevated CD28^-^ T cells may contribute to a higher risk of both articular and extra-articular complications in LORA. Conventional disease-modifying antirheumatic drugs (DMARDs) used in LORA are similar to those used in YORA. Methotrexate is often the first choice for LORA; however, the dosage should be adjusted according to renal function. Biologic DMARDs is used less frequently in LORA than in YORA, as patients with LORA may be at a higher risk for serious infections. Furthermore, patients with LORA are at an increased risk of cardiovascular disease, fragility fractures, and malignancy compared to those with YORA; they also exhibit a higher prevalence of geriatric syndrome features. Furthermore, the use of antirheumatoid drugs can influence geriatric syndromes.

## Introduction

Rheumatoid arthritis (RA) is a chronic autoimmune disease that primarily affects synovium, leading to joint damage and irreversible disability if left untreated [1]. Worldwide, a high incidence occurs between the ages of 30 and 50 years [2]. Late-onset RA (LORA), defined as onset after the age of 60 or 65 years [3, 4], represents 10-33% of all cases of RA [5]. With increasing life expectancy, the prevalence of LORA is rising, reaching approximately 2% compared to 0.5-1% for young-onset RA (YORA) [6, 7]. Whereas YORA shows a strong female predominance, with a female-to-male ratio of 3:1-4:1, LORA has a more balanced sex distribution, with a ratio closer to 2:1-1:1 [2, 8]. Clinically, LORA is more likely to present with an acute onset and preferential involvement of large joints, particularly the shoulders [9]. Patients with LORA have higher levels of erythrocyte sedimentation rate (ESR), C-reactive protein (CRP), anti-cyclic citrullinated peptide antibodies and interleukin-6 (IL-6) compared to their younger counterparts, whereas the prevalence of rheumatoid factor (RF) and the level of tumor necrosis factor-alpha (TNF-α) are lower in LORA than in YORA [10].

Aging affects both innate and adaptive immunity [11]. With aging, the innate immune system is activated, whereas the adaptive immune system tends to become dysfunctional, contributing to chronic inflammation, comorbidities, and a higher prevalence of autoimmune diseases [12]. The prevalence of comorbidities such as cardiovascular disease, malignancy, and osteoporosis is higher in patients with RA, including those with YORA, than in age-matched controls and increases further in patients with LORA [4]. These distinctions underscore the unique clinical profile of LORA and highlight the need for tailored treatment strategies.

To better understand the differences between LORA and YORA, we comprehensively review LORA’s characteristics, comorbidities, and geriatric syndromes from both immunological and therapeutic perspectives.

### Immunological difference between LORA and YORA

RA develops due to inherited genetic factors, immune system imbalances, and environmental influences. These contributing factors exhibit distinct patterns in LORA compared to YORA (Table 1).

**Table 1:** The Characteristics and Immunological features comparing late-onset rheumatoid arthritis and young-onset rheumatoid arthritis.

	LORA	YORA
**Characteristics**		
**Age of onset**	after the age of 60 or 65	30-50
**Female/male**	1:1 to 2:1	3:1 to 4:1
**Onset**	acute	gradual
**Joint**	large/proximal joints, shoulders, knees	small joints of the hands and feet
**Number of joints**	oligoarticular	polyarticular
**Clinical synptom**	resembles classical YORA	--
	resembles PMR	
	RS3PE syndrome	
**ESR/CRP**	higher	--
**Anti-CCP antibody**	higher	--
**RF**	lower	--
**IL-6**	higher	--
**TNF-α**	lower	--
**Immunological features**		
**Genetic predisposition**	HLA-DRB1*01	HLA-DRB1*04
	HLA-DRB1*01:01	HLA-DRB1*04:05
	HLA-DRB1*04:01	
	HLA-DRB1*04:05	
	HLA-DRB1*04:03	
	HLA-DRB1*14:02	
**Macrophage M1**	Increased	--
**Macrophage M2**	Decreased	--
**MerTK**	Decreased	--
**CD56^bright^**	Decreased	--
**CD56^dim^**	Increased	--
**ABCs**	Increased	--
**Telomerase activity**	Decreased	--
**CD8+ T**	Decreased	--
**CD28^-^ T**	Increased	--
**MMP-3**	Increased	--

LORA, late-onset rheumatoid arthritis; YORA, young-onset rheumatoid arthritis; ESR, Erythrocyte sedimentation rate; CRP, C-reactive protein; CCP, cyclic citrullinated peptide; RF, rheumatoid factor; IL-6, interleukin-6; TNF-α, tumor necrosis factor-alpha; Mer proto-oncogene tyrosine kinas, MerTK; ABCs, age-associated B cells

### Human Leukocyte Antigen (*HLA*) Complex in LORA and YORA

The *HLA* region contains highly polymorphic genes that encode class I and II molecules responsible for antigen presentation [13]. Genetic factors significantly influence disease susceptibility in RA, as first-degree relatives of affected individuals have a two- to five-fold increased risk compared to the general population [1]. Among the *HLA* class II alleles, *HLA-DRB1* has been identified as a significant risk factor for RA and has been widely confirmed [14]. Accumulating evidence has firmly established distinct associations of *HLA-DRB1* alleles with YORA and LORA [15]. Specifically, HLA-DRB1*04 is associated with YORA, whereas HLA-DRB1*01 is more frequently associated with LORA [15]. Hellier et al. reported a significantly lower frequency of HLA-DRB1*04 and a trend toward increased HLA-DRB1*01:01 in LORA compared to YORA [16]. In a cohort including 89 YORA patients and 329 patients with LORA, the analysis of *HLA-DRB1* allele carrier frequencies demonstrated that HLA-DRB1*04:01 was associated with susceptibility to LORA (*p* = 0.0004, odds ratio [OR] 3.11, 95% confidence interval [CI] 1.69-5.73), while HLA-DRB1*04:05 was associated with susceptibility to both YORA (*p* = 8.33 × 10^-14^, OR 5.47, 95% CI 3.47-8.61) and LORA (*p* = 2.45 × 10^-12^, OR 2.57, 95% CI 1.98-3.34) when compared to controls [15]. Moreover, the frequencies of HLA-DRB1*04:03 and HLA-DRB1*14:02 are significantly higher in patients with LORA than in patients with YORA [17].

### Differences in innate immune response between LORA and YORA

It has been widely confirmed that the dysregulation of the innate immune system is central to the pathogenesis of RA [5].

#### Macrophages

Activated macrophages (*M1*) promote joint erosion primarily by secreting pro-inflammatory cytokines such as TNF-α and IL-1. Alternatively activated macrophages (*M2*) play a role in vasculogenesis, tissue remodeling, and repair through the production of anti-inflammatory cytokines, mainly IL-10 and transforming growth factor-beta (TGF-β) [18]. However, in older individuals, macrophage function declines, with a notable reduction in phagocytic activity [19]. In patients with LORA, cytokine secretion is markedly delayed, leading to impaired responses to pathogen- and damage-associated molecular patterns. Macrophages isolated from aged humans and mice exhibit functional impairments, including weakened early immune defense and a diminished capacity to support the development of adaptive immune responses [20]. Aging also skews macrophage polarization with increased *M1* and reduced *M2* activity [19]. This imbalance impairs mesenchymal stem cell differentiation into osteoblasts, enhances osteoclastogenesis through hematopoietic stem cells, and accelerates bone resorption [18, 20]. Although no solid evidence exists supporting differences in macrophages between LORA and YORA, we suggest that such polarization shifts may contribute to more severe bone destruction in LORA. In addition to polarization, other age-related changes in macrophage biology may influence RA pathogenesis. The macrophage-expressed myeloid-epithelial-reproductive (Mer) proto-oncogene tyrosine kinase (MerTK) receptor exerts an anti-inflammatory effect by promoting the synthesis of lipid mediators, such as lipoxins and resolvins [21-23]. In older individuals, MerTK expression declines, potentially compromising the resolution of inflammation and increasing susceptibility to autoimmune diseases, including RA [24].

#### Natural killer cells

Natural killer (NK) cells are typically divided into subsets based on CD56 and CD16 expression. CD56^dim^ NK cells display potent cytotoxicity, whereas CD56^bright^ NK cells are specialized for cytokine release, such as IFN-γ and TNF-α [25]. In RA, CD56^bright^ NK cells accumulate in inflamed synovial tissues, where they serve as an abundant source of pro-inflammatory cytokines [26]. Aging alters this balance, with a relative decline in CD56^bright^ cells and expansion of the CD56^dim^ subset [27]. This age-related redistribution may contribute to lower TNF levels in LORA compared to YORA, even in the presence of heightened systemic inflammation.

### Adaptive immune response difference between LORA and YORA patients

#### B cell

B cells play an essential role in adaptive immunity [28]. With aging, immunosenescent remodeling results in a unique subset of B cells called age-associated B cells (ABCs) [29]. These cells, first identified in the spleens of aged mice, are characterized by high expression of myeloid markers, such as CD11c, CD21, and CD11b, as well as the transcription factor T-bet [30]. Unlike conventional B cells, ABCs fail to respond to B-cell receptor (BCR) and CD40 stimulation but proliferate and differentiate in response to TLR7 and IL-21 signals [31]. Therefore, ABCs may play an important role in RA [32]. They have high surface expression of molecules, such as MHC-II, CD86, and IL-13Rα1, whose levels correlate with swollen joint count, tender joints, and disease activity score 28 (DAS28) [33]. Notably, in RA, ABCs are elevated in the peripheral blood of patients with high disease activity, whereas they decline in patients who respond favorably to treatment [34]. In addition to being increased in peripheral circulation, ABCs are enriched in synovial tissue and synovial fluid [35]. This accumulation pattern has been linked to more pronounced joint erosion in LORA compared to YORA. Functionally, ABCs are regarded as plasma cell precursors; however, due to their impaired responsiveness to BCR signaling, they generally generate fewer antibodies, which may further contribute to disease progression [36].

ABCs are not only precursors of plasma cells but also active cytokine producers. In RA, they display elevated transcription of cytokines such as TNF-α, IL-17, and IL-21, establishing a pro-inflammatory signature distinct from conventional B cells and positioning them as the most inflammatory B-cell subset [37]. Furthermore, IL-6 is consistently elevated in both the synovial microenvironment and circulation of patients with RA and is markedly increased in LORA [38]. Notably, despite heightened systemic inflammation, TNF-ɑ levels tend to be lower in LORA compared to YORA, a paradox likely explained by the complex and multifactorial nature of immunosenescence that extends beyond single biomarker assessment.

#### T-cells

The function of the T-cell compartment is highly time-dependent and undergoes significant restructuring with age, as consistently reported across studies [37]. Age-related shifts in T-cell subsets are evident, with younger individuals showing distinctly different profiles compared to older individuals. Notably, a reduction in naïve CD4+ T cells has been identified as the most robust predictor of progression from anti-citrullinated peptide antibody positivity to synovitis [39].

Telomere attrition may also play a role in RA progression. Defective DNA repair mechanisms accelerate telomere loss, and telomere erosion appears to proceed more rapidly in subjects with RA than in healthy controls [37, 40]. For instance, telomere lengths in progenitor cells of patients with RA aged 20-30 years are shortened to approximately 9,000 base pairs, comparable to those of 50-60-year-old healthy individuals [40]. Age-related declines in telomerase activity further limit the ability to restore telomeres. A reduction in CD8^+^ T cells has been reported in LORA, and given the inverse relationship between CD8^+^ T cells and MMP-3, this may predispose patients to enhanced joint destruction [41]. Additionally, patients with LORA show an increase in CD28^-^ T cells, which is associated with more severe systemic disease. CD28^-^ T cells are particularly elevated in cases of extra-articular complications and are associated with an increased risk of atherosclerotic disease in RA [4].

#### Clinical presentation difference between LORA and YORA

In YORA, the disease usually begins insidiously with mild symptoms that evolve slowly over time. In contrast, LORA often presents abruptly with more acute manifestations, sometimes mimicking infectious processes. Whether this presentation reflects a primary disease phenotype or occurs secondary to chronic inflammatory changes remains uncertain [4].

The prevalence of RA is higher in females than males, with a female-to-male ratio of approximately 3:1 to 4:1 in YORA, decreasing to 1:1 to 2:1 in LORA [8]. Changes in the female hormonal environment, such as those occurring during menopause, may contribute to increased susceptibility to RA [8]. Before menopause, the ovaries produce three types of estrogens: estradiol, estrone, and estriol. Estradiol exerts an anti-inflammatory effect, whereas estrone, particularly in collaboration with TNF-α, activates the NF-κB signaling pathway and plays an important role in RA pathogenesis [42, 43]. After menopause, total estrogen levels decline, and the estrone-to-estradiol ratio increases, which may explain the loss of female predominance in LORA [43]. In men, low testosterone and high sex hormone-binding globulin (SHBG) levels are associated with an increased risk of RA [44]. A cross-sectional analysis included 3,110 male participants, including 191 patients with RA, showed that low testosterone levels (< 300 ng/dL; OR = 2.30, 95% CI: 1.65-3.21, *p* < 0.001) and elevated SHBG levels (> 57 nmol/L; OR = 1.65, 95% CI: 1.14-2.39, *p* = 0.008) were associated with a higher risk of RA. Estrogens promote humoral immunity by stimulating B-cell and Th2 responses and enhancing the survival of autoreactive lymphocytes, whereas androgens and progesterone exert immunosuppressive effects [45].

YORA typically affects the small joints of the hands and feet, less frequently involves large or proximal joints, such as the shoulders and knees, and often follows an oligoarticular pattern that can mimic polymyalgia rheumatica (PMR) [46]. Specifically, the small joints of the feet are commonly involved in YORA, whereas shoulder involvement is predominant in LORA [47]. Current evidence suggests that LORA can be classified into three distinct clinical subtypes. The first and most common subtype, accounting for approximately 70% of cases, closely resembles classical YORA and is typically RF positive [3]. The second subtype, representing approximately 25% of patients, is characterized by an acute onset, predominant involvement of large and proximal joints, and resembles PMR [48]. The third subtype is remitting seronegative symmetrical synovitis with pitting edema syndrome, marked by symmetrical synovitis and swelling of both the upper and lower extremities. Laboratory results differ between LORA and YORA. Patients with LORA have higher ESR, CRP, CCP, and IL-6, but lower RF and TNF-α compared to YORA.

#### Treatment and treatment response differences between LORA and YORA

The treatment approaches are similar for both LORA and YORA groups. However, drugs used in older patients may differ from those used in younger patients. Studies indicate that patients with LORA are more likely to begin treatment with DMARD monotherapy and GCs, but are less likely to receive combination DMARD therapy or biological drugs compared to patients with YORA.

In the early stages of LORA management, nonsteroidal anti-inflammatory drugs (NSAIDs) are usually not the first choice because older adults are more susceptible to their side effects, and NSAID use is contraindicated in most older patients with RA due to comorbidities [2]. Almost 60% of patients with LORA who initially use NSAIDs are then replaced with DMARDs [47].

GCs are less commonly used to treat LORA [47, 49]. The low-dose GC RA trial showed that low-dose prednisolone had beneficial long-term effects in older patients with established RA, with a 24% increase in patients experiencing mostly non-severe adverse effects [50]. The long-term use of GCs increases the risk of infections and cardiovascular events [50]; therefore, GCs should be used cautiously in older patients.

#### Classical DMARDs

The classical DMARDs (cDMARDs) used in the YORA can also be safely used to treat LORA. However, the pharmacokinetics and pharmacodynamics of drugs differ in older adults, and their side effect profiles should be closely monitored [3]. Chen et al. found that cDMARD use was more prevalent in YORA (35.4%) than in LORA (26.7%) in a Chinese cohort [51]. Furthermore, patients with LORA are more likely to start cDMARD monotherapy and are less likely to receive a combination of cDMARDs or biological DMARDs (bDMARDs) compared to patients with YORA [4].

Methotrexate (MTX), the anchor drug for RA, is selected as the initial cDMARD in approximately 85% of patients with LORA, and it is used more often in LORA than in YORA patients[47]. The dose of MTX can be rapidly titrated to approximately 25 mg once weekly, although lower doses are commonly used in Asian populations [52]. In addition to these regional prescription practices, patients with LORA tend to receive lower MTX doses than patients with YORA, mainly due to age-related comorbidities such as renal impairment and hepatic dysfunction, which increase the risk of drug-related toxicity [53]. The Treat-to-Target (T2T) strategy highlights the systematic and regular adjustment of therapy based on predefined targets, typically remission or low disease activity in RA [54]. Matsumoto et al. reported that among 197 patients with LORA, 70.4% of those over 75 years initiated MTX, and 34.1% and 37.1% received a bDMARD at weeks 52 and 156, respectively [55]. Importantly, comorbid conditions frequently contribute to non-adherence to T2T goals in this population [55]. The T2T strategy is effective for LORA, however, comorbidities and adverse effects make its implementation more difficult [56]. Leflunomide or sulfasalazine can be considered part of the treatment strategy for patients with contraindications to MTX [52]. However, current evidence regarding the application of leflunomide or sulfasalazine in LORA is limited, and additional research is warranted.

#### Target synthetic/biological DMARDs (tsDMARDs/ bDMARDs)

If the treatment target is not achieved using a cDMARDs strategy, bDMARDs/tsDMARDs should be added when poor prognostic factors are present [52]. Studies on the use of bDMARDs in LORA are much fewer compared to YORA, likely due to the perception that advanced age is a significant risk factor for serious adverse events, although some studies have shown no such differences [47, 57-59]. In 2019, Janus kinase inhibitors (JAKi) were considered similar to bDMARDs in terms of effectiveness and safety [52]. Large-scale randomized controlled trials of tofacitinib and baricitinib have demonstrated the efficacy of JAKi in RA [60-64]. Compared to YORA, patients with LORA receiving tofacitinib, a JAKi (5 or 10 mg daily), have a similar remission response but a higher rate of serious adverse events and infections [60, 61]. Baricitinib has been demonstrated to have similar efficacy in LORA (≥ 65 years) and YORA, but with higher rates of serious adverse events and serious infections at doses of 2 mg and 4 mg once daily. The most common serious infections include pneumonia, herpes zoster, urinary tract infections, and venous thromboembolism [62, 63]. Based on data from the ORAL Surveillance trial, patients with RA aged ≥ 50 years have significantly higher rates of cardiovascular events [65]. Further post-hoc analysis of the data revealed that age of ≥ 65 years increased the risk of cardiovascular events, malignancies, venous thromboembolism, and serious infections [66]. Nevertheless, consistent evidence supports the idea that infections are more frequent in LORA than in YORA [60-63, 65]. Therefore, infection prevention should be a high priority for patients with LORA.

Real-world data indicate that TNF inhibitors (TNFi) have similar therapeutic effects in LORA and YORA in terms of clinical response, although functional outcomes may differ due to LORA-related comorbidities. A Japanese study demonstrated that the efficacy and safety of golimumab in patients with LORA aged ≥ 75 years were comparable to those in younger patients [67]. In addition, treatment-naïve patients with LORA have a superior response to TNFi compared to patients with YORA [68]. Evidence also supports the cardioprotective effect of TNFi, with patients initiating TNFi treatment experiencing a significant reduction in composite cardiovascular events compared to those treated with MTX or other cDMARDs [69], particularly among individuals aged ≥ 65 years [70]. However, the use of TNFi in LORA is associated with an increased risk of serious infections, including bacterial infections, tuberculosis, *Pneumocystis jirovecii* pneumonia, systemic fungal infections, cytomegalovirus, and herpes zoster. This risk does not appear to differ significantly among adalimumab, etanercept, and infliximab [71, 72]. Currently, no specific dosage adjustments are recommended for TNFi in LORA. Given the heightened risk of infection, careful monitoring and individualized risk-benefit assessment are essential when prescribing TNFi to elderly patients. Abatacept has been approved for the treatment of RA and inhibits T-cell activation by binding to CD80 and CD86 on antigen-presenting cells, thereby reducing downstream T- and B-cell responses [73]. The efficacy and safety of abatacept have been proven in patients with active disease and inadequate response to MTX or biologic DMARDs [74]. In LORA (mainly aged ≥ 65 years), abatacept appears to provide greater cardiovascular protection compared to TNFi [69]. The efficacy of IL-6 receptor inhibitors (IL-6Ri) may be slightly lower in LORA [2]. However, a study in the United Kingdom showed that patients with LORA who were treated with IL-6Ri had a higher risk of infection [2].

Studies have compared the retention rates and tolerability of b/tsDMARDs in patients with LORA; however, the findings remain inconsistent, and no definitive conclusions can be drawn on which agent is best tolerated. Current evidence suggests that abatacept and IL-6Ri may be effective therapeutic options for the treatment of LORA. Kubo et al. revealed that abatacept and IL-6Ri had the highest continuation rates in patients with LORA (≥ 75 years) due to their adequate efficacy, suggesting their potential suitability for this specific elderly patient population [75]. A real-world study from Italy, including 1,221 patients with LORA treated with biologics or tsDMARDs (29.6% TNFi, 24.8% abatacept, 16.3% IL-6Ri, 24.9% JAKi, and 4.4% rituximab), also highlighted treatment durability.

**Table 2:** Overview of Disease-Modifying Antirheumatic Drugs in Late-Onset Rheumatoid Arthritis.

Category	Class	Drug
DMARDs	cDMARDs	Methotrexate
		**Leflunomide**
		**Sulfasalazine**
tsDMARDs/bDMARDs	JAKi	Tofacitinib
		**Baricitinib**
		**Upacitinib**
	TNFi	Etanercept
		**Adalimumab**
		**Infliximab**
		**Certolizumab**
		**Abatacept**
	IL-6Ri	Tocilizumab
		**Sarilumab**

DMARDs, disease-modifying antirheumatic drugs; cDMARDs/tsDMARDs/bDMARDs, classcial/target synthetic/biological DMARDs; JAKi, Janus tyrosine kinase inhibitor; TNFi, tumor necrosis factor inhibitors; IL-6Ri, interleukin-6 receptor inhibitor

Abatacept showed significantly higher drug retention compared with TNFi (*p* = 0.02), JAKi (*p* < 0.001), and IL-6Ri (*p* < 0.001). TNFi demonstrated higher retention than JAKi (*p* = 0.013). Loss of efficacy was the primary cause of discontinuation [76]. However, contrasting findings have been reported by Jinno et al., who evaluated biological drug retention in Japanese patients with LORA. The cohort included 572 patients who received 835 treatment courses (314 TNFi, 175 IL-6Ri, 228 cytotoxic T-lymphocyte-associated antigen 4 immunoglobulin blockers, and 118 JAKi) [77]. The results showed that IL-6Ri and JAKi had longer treatment retention and lower discontinuation rates due to a lack of efficacy. However, discontinuations due to adverse events were more frequent in the JAKi group. Evidence from these studies indicates that advanced age does not contraindicate the use of DMARDs. Nonetheless, the potential risks of bDMARDs and tsDMARDs in LORA require careful assessment, emphasizing the importance of individualized treatment strategies. The DMARDs used in LORA are listed in Table 2.

#### Comorbidities

Common comorbidities in patients with LORA are shown in Table 3.

**Table 3:** Overview of comorbidities and geriatric syndrome in Late-Onset Rheumatoid Arthritis.

Category	Examples
Comorbidities	Cardiovascular disease
	**Fragility fracture**
	**Malignancies**
Geriatric syndrome	Sarcopenia
	**Cognitive impairment**
	**Frailty**

#### Cardiovascular disease

Compared to the general population, patients with RA have a twofold higher risk of cardiovascular disease [78]. Studies have reported more cardiovascular events in patients with LORA compared to those with YORA [46]. This might be due to the presence of classical cardiovascular risk factors, such as hypertension, dyslipidemia, and obesity, which are associated with aging [79, 80]. Furthermore, aging and RA can independently increase the risk of cardiovascular disease, making it difficult to determine the extent to which RA treatment contributes to cardiovascular complications [10]. In addition, Patients with LORA present higher levels of inflammatory biomarkers compared to those with YORA, including ESR/CRP, anti-CCP, and IL-6, which are closely involved in the pathogenesis of cardiovascular diseases. Furthermore, GCs and NSAIDs may further amplify cardiovascular risk by promoting inflammation and atherosclerosis, particularly in LORA [81]. Therefore, the combined effects of aging, RA itself, systemic chronic inflammation, and certain medications may contribute to a higher cardiovascular burden in LORA compared to YORA [82].

#### Fragility fracture

Fragility fractures are defined as fractures resulting from low-energy trauma, typically a fall from standing height or lower, which would not ordinarily cause a fracture in healthy bone [83]. The prevalence of fragility fractures rises markedly with age, and global meta-analyses have reported an overall incidence of 15.31 (95% CI, 10.43-22.47) per 1,000 person-years [84]. Patients with RA have approximately twice the risk of fragility fractures as age- and sex-matched controls [85]. In older adults, fragility fractures are associated with a substantially higher risk of disability and poor outcomes [86]. The most common sites include vertebral, hip, forearm, and proximal humeral fractures, with incidence rates of 7.51 (95% CI, 3.27-17.23), 4.33 (95% CI, 2.26-8.27), 3.40 (95% CI, 2.27-5.10), and 1.86 (95% CI, 1.36-2.53) per 1,000 person-years, respectively [84]. In RA, chronic inflammation and immune dysregulation contribute to bone fragility [87]. In the elderly population, increased *M1* and reduced *M2* activity result in decreased osteoblast and increased osteoclast formation, both of which accelerate bone resorption [18, 20]. These mechanisms may partially explain the higher prevalence of fragility fractures in patients with LORA. Exposure to GC is a well-known risk factor. Higher daily or cumulative doses and long-term use may increase the risk of fragility fractures [88]. MTX is an anchor drug used in RA therapy that can increase the risk of fragile fractures, a condition known as methotrexate osteopathy [89]. In one study of 33 patients (mean age, 67.3 years; mean weekly MTX dose, 20 ± 5.9 mg; mean treatment duration, 10.7 ± 6.2 years), continuation of MTX after the first insufficiency fracture was associated with a significantly higher risk of subsequent fractures [89]. Therefore, MTX should be used with particular caution in patients with LORA who present with an initial fracture, and discontinuation should be considered to minimize the risk of future fractures.

#### Malignancies

Cancer is the second leading cause of death in patients with RA, and the overall risk of malignancy is higher than that in the general population [9]. In patients with RA, the incidences of lymphoma (Standardized incidence ratio [SIR], 2.46), lung neoplasia (SIR, 1.64), and melanomas (SIR, 1.23) are elevated [90]. In addition to long-term chronic inflammation and defective DNA repair, immune senescence may contribute to this risk [4]. It appears that DMARDs do not increase the risk of cancer [91]; however, the risk of melanoma may increase slightly [91]. Furthermore, when treating patients with a history of cancer, JAKi and abatacept should be used with caution, as they may influence the risk of recurrence [91]. Aging and cancer are closely related and share common pathogenic mechanisms. Several hallmarks of aging also contribute to carcinogenesis and tumor progression, but some of the molecular and cellular characteristics of aging may reduce the probability of lethal cancer development [92]. However, reports on the rates of malignant cancer in LORA and YORA are limited.

#### Geriatric syndrome

Geriatric syndrome (GS) is a multifactorial health condition that makes older people vulnerable to health or social challenges. Frailty is a prime example [93]. The common manifestations of GS include frailty, sarcopenia, dementia, urinary incontinence, malnutrition, sensory impairment, and falls [94]. Generally, due to geriatric syndromes, polypharmacy, and multimorbidity, older patients are trapped in a downward spiral [93], which may ultimately result in new health issues. For example, patients with RA may present with features such as sarcopenia [95], fatigue, and low physical activity [96], especially when the disease is poorly controlled [97]. Furthermore, higher disease activity, as measured by DAS28-ESR, is a risk factor for sarcopenia in patients with RA [98].

#### Sarcopenia

Sarcopenia is the accelerated loss of skeletal muscle mass and function that is not exclusively associated with advancing age [99]. It is associated with a range of adverse health outcomes, including physical disability, insulin resistance, falls, frailty, and increased mortality [100]. Approximately 25% of patients with RA may be affected by sarcopenia [101]. In these patients, reduced muscle mass and strength compromise physical performance and contribute to higher levels of disability [100]. Sarcopenia is closely associated with RA, potentially due to chronic inflammation mediated by cytokines such as TNF, IL-6, and IFN-γ, which disrupt muscle homeostasis in RA [100]. Additionally, skeletal muscle mass decreases by 3% per year after age 60, which may explain why sarcopenia is more prevalent in LORA than YORA [98].

Several studies have investigated the prevalence and risk factors for sarcopenia in patients with RA. Myroslava et al. enrolled 138 patients with inflammatory arthritis, including 17 with YORA (median age 33.0 years). Using the European Working Group on Sarcopenia in Older People (EWGSOP2) criteria, they reported a sarcopenia prevalence of 59% in this cohort [102]. In a prospective registry of 76 middle-aged patients with RA (mean age, 56.5 years), the prevalence of sarcopenia assessed according to EWGSOP criteria was 10.5%. Multivariate analysis further demonstrated that age ≥ 60 years was significantly associated with an increased risk of sarcopenia (OR = 3.3; 95% CI, 1.4-7.7; *p* = 0.005) [103]. Katchamart et al. reported a lower rate of sarcopenia in patients with LORA. This study included 182 patients with LORA, with a mean age of 59.2 ± 10.2 years. Among them, 48 patients (26.4%) were classified as having sarcopenia according to the 2019 Asian Working Group criteria. Patients with sarcopenic were significantly older (mean age, 62.8 ± 1.4 vs. 57.8 ± 0.9 years; *p* = 0.003) and had higher disease activity, as reflected by DAS28 (2.82 ± 0.86 vs. 2.53 ± 0.80; *p* = 0.04) and ESR (46.5 [21-58.5] vs. 30 [16-44]; *p* = 0.01) [104]. In LORA, patients with sarcopenia tended to be older and less likely to be in remission compared to their non-sarcopenic counterparts, although these differences were not statistically significant (*p* = 0.072) [98].

Regarding treatment, a meta-analysis evaluated the association between antirheumatic drugs and sarcopenia in both middle-aged patients with RA and those with LORA, including a total of 2,240 participants (mean age, 47.7 ± 5.5 years). The analysis showed that GC use was positively associated with sarcopenia (OR = 1.46), whereas cDMARDs were inversely associated with sarcopenia (OR = 0.70). No significant association was observed for b/tsDMARDs (OR = 0.83) in either middle-aged patients with RA or those with LORA [105].

#### Cognitive impairment

Chronic systemic inflammation observed in RA predisposes patients to cognitive dysfunction, which is associated with increased functional limitation, depression, and pain [4]. Recent evidence indicates that RA is associated with an increased risk of cognitive decline [106]. Peripheral inflammation and cytokines can alter brain metabolism [107]. Furthermore, inflammation accelerates atherosclerosis, which may contribute to cognitive impairment in RA [108]. Persistent pain can also affect brain circuitry and disturb cognition and behavior. MTX and GCs may influence cognitive impairment, but the evidence remains controversial, and the underlying mechanisms are not well understood [108, 109]. MTX and GCs dampen systemic inflammation and may lower the risk of cognitive impairment [108]. In contrast, MTX is associated with cognitive decline, confusion, and mood changes. GCs can impair memory and hippocampal function, potentially increasing the risk of cognitive decline [109].

The prevalence of dementia is higher in patients with LORA compared to YORA. A cross-sectional study of 98 participants, including 65 LORA and 33 YORA cases, showed that patients with LORA were significantly more prone to cognitive impairment (*p* = 0.002) [110]. A registry-based cohort study examined the association between LORA and cognitive decline. The study enrolled 1,685 patients with LORA (mean age 79.9 ± 6.7 years) and 5,055 non-RA participants (mean age of 80.1 ± 7.5 years). Compared to non-RA patients, LORA was significantly associated with faster cognitive decline (*p* = 0.001) [111]. Therefore, both age and RA can cause cognitive impairment.

#### Frailty

Frailty is increasingly recognized as a critical determinant of outcomes in older adults with RA, particularly LORA [112]. The presence of frailty not only exacerbates disability and vulnerability to adverse health events but also complicates disease management and treatment decision-making. Frailty assessment in RA is challenging because of the wide range of available instruments and the overlap between frailty criteria and RA-related symptoms. Frailty assessment tools are generally classified into two types: the physical frailty phenotype, which includes Adapted Fried’s Criteria, Ageing and Retirement in Europe Frailty Instrument (SHARE-FI), and the Study of Osteoporotic Fractures Index; and frailty based on the deficit accumulation model [113], which includes the Comprehensive Rheumatologic Assessment of Frailty (CRAF), the Kihon Checklist (KCL), and the 5-Item Frailty Score. The frailty phenotype [114], SHARE-FI [115, 116], KCL, and CRAF have been used to assess frailty in patients with RA [112]. Salaffi et al. confirmed that KCL, CRAF, and SHARE-FI are appropriate tools for defining and evaluating frailty in RA [117]. The CRAF demonstrated robust validity and superior discriminatory power compared to the SHARE-FI [118].

Multiple frailty assessment tools have been used to evaluate the impact of RA treatment on frailty. However, no standardized approach has been established, and it remains unclear which tool is most suitable for guiding therapeutic decisions. The 5-Item Frailty Screening Index has been linked to disease activity, disability, and health-related quality of life. Furthermore, NSAIDs use (OR 1.59, 95% CI 1.23-1.98) and MTX use (OR 0.75, 95% CI 0.60 -0.94) were significantly (*p* < 0.05) associated with frailty [119]. In addition, Sobue et al. used the KCL tools and demonstrated that frailty is a significant factor contributing to MTX discontinuation due to adverse events, highlighting the need for careful monitoring of frail patients with RA receiving MTX [120]. Singh et al. used a Claims-based Frailty Index to investigate frailty and serious infections in patients with YORA (age < 65 years) who initiated b/tsDMARDs [121]. Among 57,980 patients (mean age 48.1 years), 6% were classified as frail. Frailty increased the risk of infections across TNFi [hazard ratio (HR) 1.5, 95% CI 1.2-1.9], non-TNFi biologics (HR 1.2, 95% CI 1.0-1.4), and JAKi (HR 1.4, 95% CI 1.0-2.0), highlighting the importance of frailty assessment in RA, not just LORA.

## Conclusion

LORA, a distinct subtype of RA, differs significantly from YORA in pathogenesis and clinical features. These differences are primarily characterized by the complex impact of immunosenescence on disease progression and by an increased prevalence of comorbidities. Therefore, managing LORA requires addressing the individualized needs of older patients, optimizing medication choices, and emphasizing the prevention and management of comorbidities. Further research into the mechanisms underlying LORA and evaluation of treatment efficacy will help develop more effective and targeted therapeutic strategies, ultimately improving patient prognosis and quality of life.
